# Stretching of the Back Improves Gait, Mechanical Sensitivity and Connective Tissue Inflammation in a Rodent Model

**DOI:** 10.1371/journal.pone.0029831

**Published:** 2012-01-06

**Authors:** Sarah M. Corey, Margaret A. Vizzard, Nicole A. Bouffard, Gary J. Badger, Helene M. Langevin

**Affiliations:** 1 Department of Neurology, University of Vermont, Burlington, Vermont, United States of America; 2 Department of Anatomy and Neurobiology, University of Vermont, Burlington, Vermont, United States of America; 3 Department of Medical Biostatistics, University of Vermont, Burlington, Vermont, United States of America; 4 Department of Movement and Rehabilitation Science, University of Vermont, Burlington, Vermont, United States of America; 5 Neuroscience Graduate Program, University of Vermont, Burlington, Vermont, United States of America; University of Cincinnatti, United States of America

## Abstract

The role played by nonspecialized connective tissues in chronic non-specific low back pain is not well understood. In a recent ultrasound study, human subjects with chronic low back pain had altered connective tissue structure compared to human subjects without low back pain, suggesting the presence of inflammation and/or fibrosis in the low back pain subjects. Mechanical input in the form of static tissue stretch has been shown *in vitro* and *in vivo* to have anti-inflammatory and anti-fibrotic effects. To better understand the pathophysiology of lumbar nonspecialized connective tissue as well as potential mechanisms underlying therapeutic effects of tissue stretch, we developed a carrageenan-induced inflammation model in the low back of a rodent. Induction of inflammation in the lumbar connective tissues resulted in altered gait, increased mechanical sensitivity of the tissues of the low back, and local macrophage infiltration. Mechanical input was then applied to this model as *in vivo* tissue stretch for 10 minutes twice a day for 12 days. *In vivo* tissue stretch mitigated the inflammation-induced changes leading to restored stride length and intrastep distance, decreased mechanical sensitivity of the back and reduced macrophage expression in the nonspecialized connective tissues of the low back. This study highlights the need for further investigation into the contribution of connective tissue to low back pain and the need for a better understanding of how interventions involving mechanical stretch could provide maximal therapeutic benefit. This tissue stretch research is relevant to body-based treatments such as yoga or massage, and to some stretch techniques used with physical therapy.

## Introduction

Although manual and movement-based therapies utilizing tissue stretch have shown some therapeutic benefits in clinical trials of low back pain [1], [2], [3], [4], [5], [6], [7], [8] the mechanisms of these treatments and their underlying pathological substrates are poorly understood. Recently, lumbar paravertebral soft tissues including non-specialized connective tissues have emerged as potentially important components in the pathophysiology of low back pain [9], [10]. Ultrasound imaging has revealed that altered thoracolumbar connective tissue thickness and echogenicity are associated with chronic low back pain, suggesting the presence of inflammation or fibrosis [10]. Nonspecialized connective tissues in the low back of rodents have intrinsic sensory innervation [11], [12], [13] and animal models show that inflammation of other types of connective tissues can be involved in the persistence of pain [14], [15], [16].

In addition to its potential role in chronic pain, an important characteristic of connective tissue is its responsiveness to mechanical stimulation [17], [18]. In particular, recent evidence suggests that low amplitude static (non-cyclical) stretching may have beneficial antifibrotic [19] and antiflammatory effects [20]. The goal of this study was to investigate whether gentle stretching of tissue *in vivo* could reduce inflammation within the connective tissues of the low back. We first developed a novel model of non-specialized connective tissue inflammation in the rat characterized by macrophage infiltration, increased local mechanical sensitivity as well as impaired gait. We then used this model to test the hypothesis that *in vivo* stretching of the back twice a day for 12 days improves gait, local tissue inflammation and mechanical sensitivity.

## Methods

### Ethics statement

All procedures were carried out in accordance with the Guide for the Care and Use of Laboratory Animals of the National Institutes of Health. All methods for this study were approved by the University of Vermont Institutional Animal Care and Use Committee (Approval number 09-065.)

### Mechanical Sensitivity Testing with Von Frey Filaments

To evaluate changes in mechanical sensitivity, we first conducted a full battery of testing with a pilot group of animals (n = 8) to establish the average percent response to each filament (0.16 g–6 g filaments) on the low back. From the pilot testing, the 0.16 g, 1 g and 4 g filaments elicited average responses of 0%, 19.4%, and 70% respectively. Because the 1 g filament elicited a moderate response we focused on this filament as it was in the optimal range to measure changes in mechanical sensitivity. We also used the 0.16 g filament and the 4 g filaments as low and high response controls. Measurements were taken at baseline (before injection), 1 week and 2 weeks. The back of each rodent was shaved one day prior to Von Frey filament testing. After shaving, a permanent marker was used to draw a line down the middle of the back indicating the location of the spine, and two transverse lines were drawn at the base of the ribcage and greater trochanter of the hip respectively. Half way between the transverse lines, (corresponding to the location of the L3 vertebra), points were marked bilaterally 1 cm lateral to the spine to mark the location of the injection site and 2 cm circles centered on these points were drawn to define an area for Von Frey testing. On the day of testing, animals were placed in a clear plastic chamber and filaments were applied to the right and left side of the back in the designated 2 cm circle. Utilizing a testing protocol and response criteria similar to that of Millecamps et al. (2004), each filament was applied for 2–3 seconds with a brief rest period (a few seconds) between each stimulus. When testing filaments on the back, a positive response to stimulation was considered to be twitching of the skin, change of body position or escape [21]. Freezing behavior was not recorded as a response. The filaments were applied to each side of the back 10 times and the number of responses recorded and multiplied by 10 was used to establish a percent response per animal [22], [23].

### Model of inflammation

**Figure 1:**
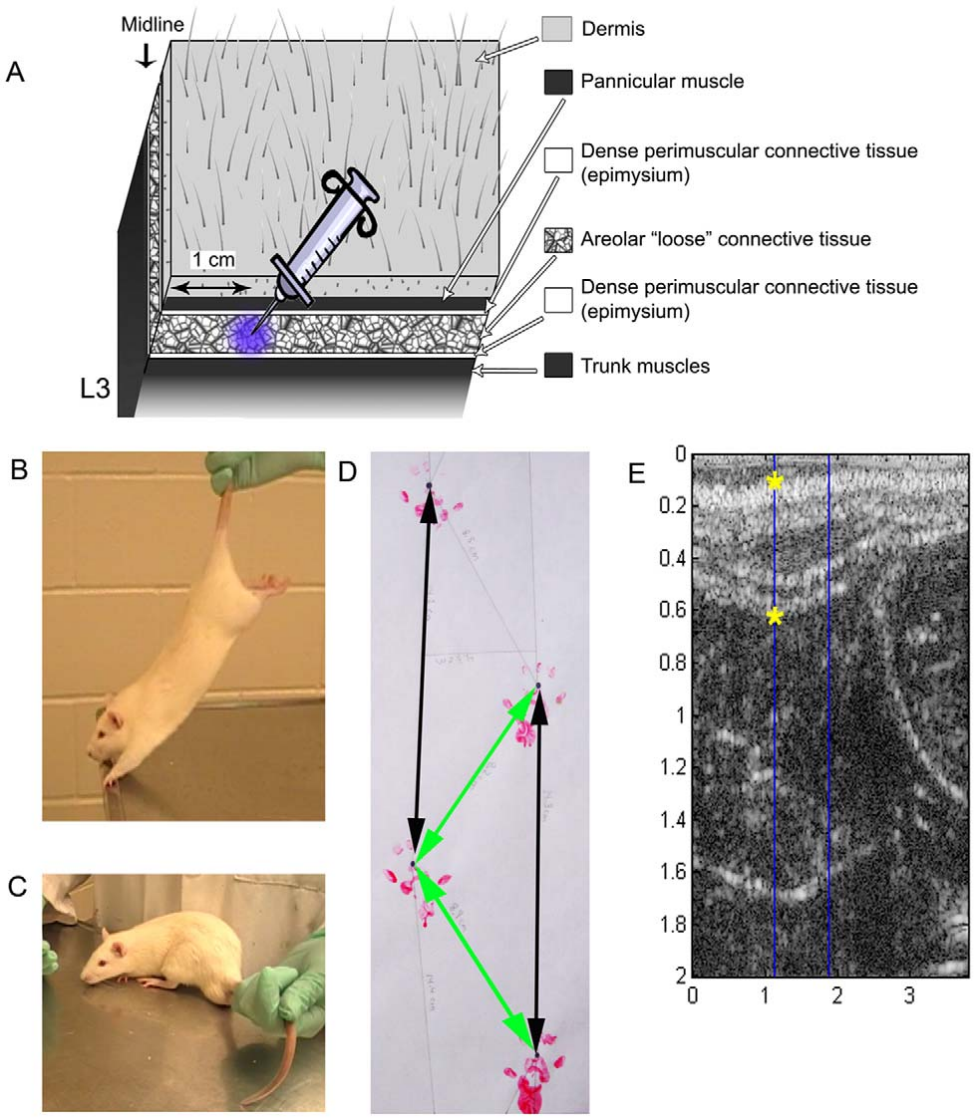
Methods for injection, stretch technique, sham technique, gait testing and ultrasound measurements. A: Cartoon illustrating the tissue layers in the low back of the rodent from the dermis to the deep back muscles. The syringe indicates the location 1 cm lateral of L3 where carrageenan or vehicle was injected. B: I*n vivo* stretch technique showing animals held by the tail and lifted. C: Sham technique (to account for the additional handling associated with stretch) showing animal held by the tail but not lifted. D: Method used for gait analysis. The distance, in centimeters, between ipsilateral walking tracks represents the stride length (black arrows). The diagonal distance between consecutive right and left walking tracks produces the intrastep distance (yellow arrows). E: Ultrasound imaging tissue thickness measurements (indicated by yellow stars).

Adult male Wistar rats (n = 36) were injected with either 100 µl of 3% carrageenan (Sigma, St. Louis, MO) or vehicle (saline) unilaterally into the subcutaneous connective tissues of the lower back under 2% isoflurane anesthesia. Animals were randomized by side of injection (right or left) and to 1 of 5 groups: vehicle/no treatment, vehicle/stretch, carrageenan/no treatment, carrageenan/sham or carrageenan/stretch. The back of each rodent was shaved and marked one day prior to injection for baseline Von Frey filament testing and to identify the injection location (see above). With the exception of the pannicular muscle, the rodent tissue structure is similar to human (Figure 1A). Injections were delivered into the subcutaneous connective tissues (the natural cleavage plane) at a point 1 cm lateral of the spine (Figure 1A). At two weeks, all animals were euthanized with 5% isoflurane anesthesia followed by decapitation. The two week time point has been described by others as demonstrating chronicity of pain and/or inflammation in rodent models [24], [25].

### Stretch and Sham technique

The *in vivo* stretch technique was adapted from the method of Bouffard et al. (2008). Animal handling was initiated for approximately five minutes per animal prior to injection. Stretch or sham intervention started two days after injection and was continued for 12 days. For stretching, animals were removed from their cage and placed on a stainless steel table. The animal was lifted gently by the tail until reaching an approximately 45° angle with the table. Once partially suspended by the tail, the animals were pulled slowly backwards and this movement encouraged the rats to grab onto the edge of the table with their front paws (Figure 1B). To encourage the animals to stretch the full length of their body, gentle pressure was applied to their hind paws to encourage them to extend their back legs away from their body (Figure 1B). Each animal was held in this position for 5 minutes in the morning and afternoon on the first day, and for 10 minutes in the morning and afternoon on each subsequent day for a total of 12 days of treatment. Animals were not stretched on the day of euthanasia to limit the effect of acute stretch on the tissue. For the sham procedure, animals were removed from their cage and placed on the same stainless steel table. Animals were handled and held by the tail in the same manner as the stretch animals, but were not lifted by the tail or stretched (Figure 1C). After completing a first set of experiments (n = 4 per condition), we added a carrageenan/sham group (n = 4) to the second set of experiments so that the sham treatment could control for the effects of additional handling and time outside of the cage.

### Open Field Testing

To establish whether the carrageenan inflammation model had an effect on activity and mobility, we measured each rat's overall activity, time spent in center of the open field, and rearing behavior at the two week time point (day 14). Each animal was placed in the center of a 45 cm×45 cm×45 cm clear plastic box and permitted to move freely for 10 minutes while being videotaped. A gridline was marked on the bottom of the box, creating 9 cm×9 cm squares. A blinded observer recorded the overall activity, center time and number of rears for each animal. Overall activity was measured as the total number of gridlines crossed. Time spent in the center was accumulated each time all four paws enter the center 9 squares. Each rear was counted as a separate event beginning when the front paws both leave the ground and ending when both paws return to the ground.

### Gait testing

Walking tracks were gathered from each rat [26] at the two week time point (day 14) by applying non-toxic finger paint to both hindpaws. Each animal was permitted to walk through a (90 cm×8.5 cm×15 cm) plastic enclosure with white paper under their feet at least 2 times to allow for the collection of a straight line of walking tracks. Animals were allowed to walk freely without external stimulus or inducement through the plastic enclosure. A minimum of 8 walking tracks (4 paw prints on each side) was obtained from each rat. However, when more than 8 walking tracks were collected all values were included in calculating the averages. Measurements were made from the base of the second toe on each hindpaw and calculated in centimeters (cm) by an individual blinded to treatment group. Stride length was calculated as the distance from one hindpaw to the next ipsilateral hindpaw print (Figure 1D, black arrows). Intrastep distance was calculated as the diagonal distance between sequential right and left tracks (Figure 1D, green arrows) [27].

### Ultrasound imaging and analysis

Immediately after euthanasia, ultrasound images were collected from the right and left sides of the low back with a Terason 3000 scanner using a linear array probe at 10 MHz. Images were taken 1 cm lateral and parallel to the spine with the caudal end of the probe positioned on the iliac crest for reproducibility. This method is adapted from a human study [10] where similar tissues were imaged. Tissue thickness measurements were calculated in centimeters from the top (superficial) of the dermis to the deep back muscles (Figure 1E, yellow stars). Tissue thickness was calculated by importing ultrasound images into Matlab (Simulink; Natick, MA) where all measurements were made by a blinded investigator. Intra- and inter-rater reliability was carried out by two individuals. For inter-rater and intra-rater reliability assessment of tissue thickness measurements in the rat, two observers each acquired three ultrasound images sequentially at the same tissue location in the manner described above.

### Histology and Immunofluorescence

After euthanasia, a tissue block was excised from the lumbar region of the back lateral to L3 (including all tissues from the dermis, connective tissues and deep spinal muscles). Tissue blocks were fixed in 4% paraformaldehyde for 24 hours and embedded in paraffin. Tissue sections were cut on a microtome at 6 µm. Routine histological methods were used for hematoxilin and eosin (H+E) staining [28]. All tissues from each set of experiments were processed at the same time, minimizing processing induced differences in tissue. For immunofluorescence staining of macrophages, paraffin sections were baked at 60°C for one hour, rehydrated through xylene and graded alcohols (Sigma) and washed in phosphate buffered saline (PBS; Sigma;St. Louis, MO). Tissues were blocked and permeabilized in PBS with 10% normal goat serum (Vector labs, Burlingame, CA) and 0.5% Triton X-100 (Sigma) (PBGT) for 30 minutes before the addition of the anti-rat CD68 primary antibody (ED1; Serotec; Raleigh, NC, 1∶1000) overnight at 4°C. After washing in PBS, tissue sections were incubated with goat anti-mouse Alexa 488 (Invitrogen; Carlsbad, CA). Slides were again rinsed and coverslipped with Citifluor mounting medium (Citifluor Ltd; London, UK). For imaging, slides were coded and marked at the point 1 cm lateral of the midline (site of injection). At that distance from the midline, a field was imaged immediately deep to the subcutaneous muscle. An Olympus BX50 microscope and Magnafire software (Optronics; Goleta, CA) were used to collect images. Metamorph imaging analysis software (Molecular Devices; Sunnyvale, CA) was used to quantify macrophage expression by applying the same threshold (excluding background staining) to each image and quantifying the number of positively staining pixels above threshold. Results are expressed as percent staining area (staining area over total imaged area).

### Statistical Methods and Data Analysis

Two-factor analyses of variance and covariance were used to compare outcome measures across experimental conditions. In addition to the factor representing the five experimental conditions, the statistical model included a second fixed factor representing the two sets of experiments performed with and without the sham condition. Analysis relating to Von Frey testing utilized animal's baseline values as a covariate when evaluating the two week time point. Pairwise comparisons between treatment means were performed using Fisher's LSD procedure conditional on a significant overall F-test. All means presented represent least square means which adjust for potential differences across the two sets of experiments. Means presented for Von Frey testing are also adjusted for baseline values. Intraclass correlation coefficients were used to estimate the intra-rater and inter-rater reliability of ultrasound measurements of tissue thickness. All analyses were performed using SAS Statistical Software Version 9.2 (SAS Institute, Cary NC). Statistical significance was determined based on α = 0.05. Data were output into graphs with GraphPad Prism Version 5 (Graphpad Software; San Diego, CA).

## Results

All experiments were carried out concurrently and data are presented from all 5 experimental groups: vehicle/no treatment, vehicle/stretch, carrageenan/no treatment, carrageenan/sham and carrageenan/stretch. To evaluate the inflammation model, results from the carrageenan/no treatment group are compared to the vehicle/no treatment group. To evaluate the stretch intervention, the carrageenan/stretch group is compared to both the carrageenan/no treatment and the carrageenan/sham groups. All results from the vehicle/stretch group were found to be similar to the vehicle/no treatment group and thus these individual results are not described further.

### Gait Testing

**Figure 2:**
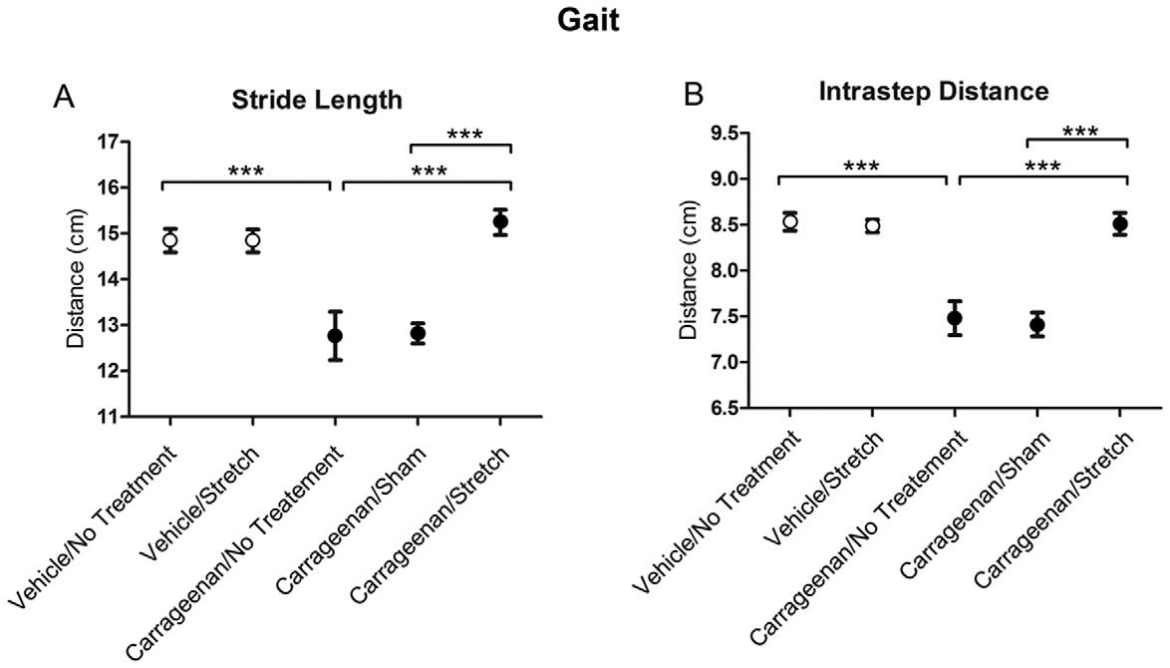
Gait analysis measurements. Stride length (A) and intrastep distance (B) across experimental groups. Open and closed circles respectively denote the average measurements for vehicle and carrageenan groups. Standard errors (S.E.) are indicated with error bars. N = 4 carrageenan/sham, N = 8 all other groups. (***p<.001).

To evaluate changes in gait, the stride length, intrastep distance and stride width were measured by examining walking tracks from each animal on day 14 of the experiment, one day prior to euthanasia. Average stride length and intrastep distance were significantly different among the experimental conditions (p<.001 for both variables). The carrageenan/no treatment group demonstrated a significantly shorter stride length (p<.001) and intrastep distance (p<.001) compared to the vehicle/no treatment group (Figure 2A,B). Stretching for 12 days increased both stride length and intrastep distance in the carrageenan/stretch group to levels similar to the vehicle condition (Figure 2 A,B). The carrageenan/sham intervention, however, was not significantly different from carrageenan/no treatment. Stride width did not differ across the five experimental conditions (p = .31) (Data not shown).

### Open Field Test

An open field test (10 minutes) was used to evaluate the overall activity and mobility levels for all experimental groups. The goal of this test was to determine if there was sickness behavior associated changes in activity due to the carrageenan injection. With open field testing, mean activity and mobility levels were similar across conditions (p = .64) (Data not shown).

### Mechanical stimulation testing with Von Frey filaments

**Figure 3:**
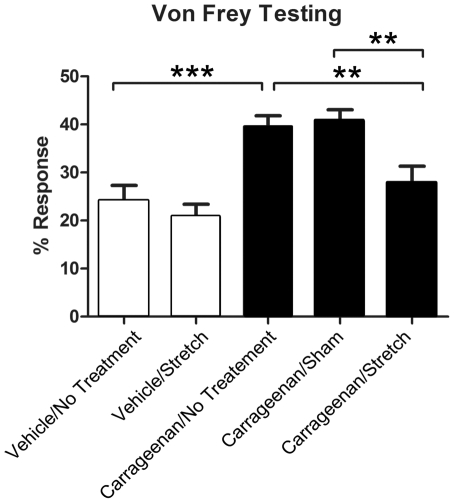
Von Frey filament testing for mechanical sensitivity in the low back. Bar graphs illustrate the mean percentage response out of ten trials for each experimental group at two weeks with the 1 g filament. Open and closed bars respectively represent means ±S.E for vehicle and carrageenan groups. For each group, the average response at two weeks was adjusted for baseline Von Frey measurements. N = 4 carrageenan/sham, N = 8 all other groups. (**p<.01, ***p<.001).

Von Frey filament testing was used to measure changes in local mechanical sensitivity in the tissues of the low back. At one week, increased sensitivity was observed in all inflammation groups (compared to vehicle) but there were no statistically significant differences between the inflammation, inflammation/sham and inflammation/stretch groups (data not shown). At the two week time point, mechanical sensitivity to the 1 g filament differed significantly across conditions (ANCOVA adjusted for baseline p<.001, Figure 3). The carrageenan/no treatment group demonstrated increased mechanical sensitivity compared to the vehicle/no treatment group (p<.001). This increased mechanical sensitivity was ameliorated in the carrageenan/stretch compared to both the carrageenan/no treatment group (p<.01) and the carrageenan/sham group (p<.01) (Figure 3).

### Ultrasound imaging and analysis

**Figure 4:**
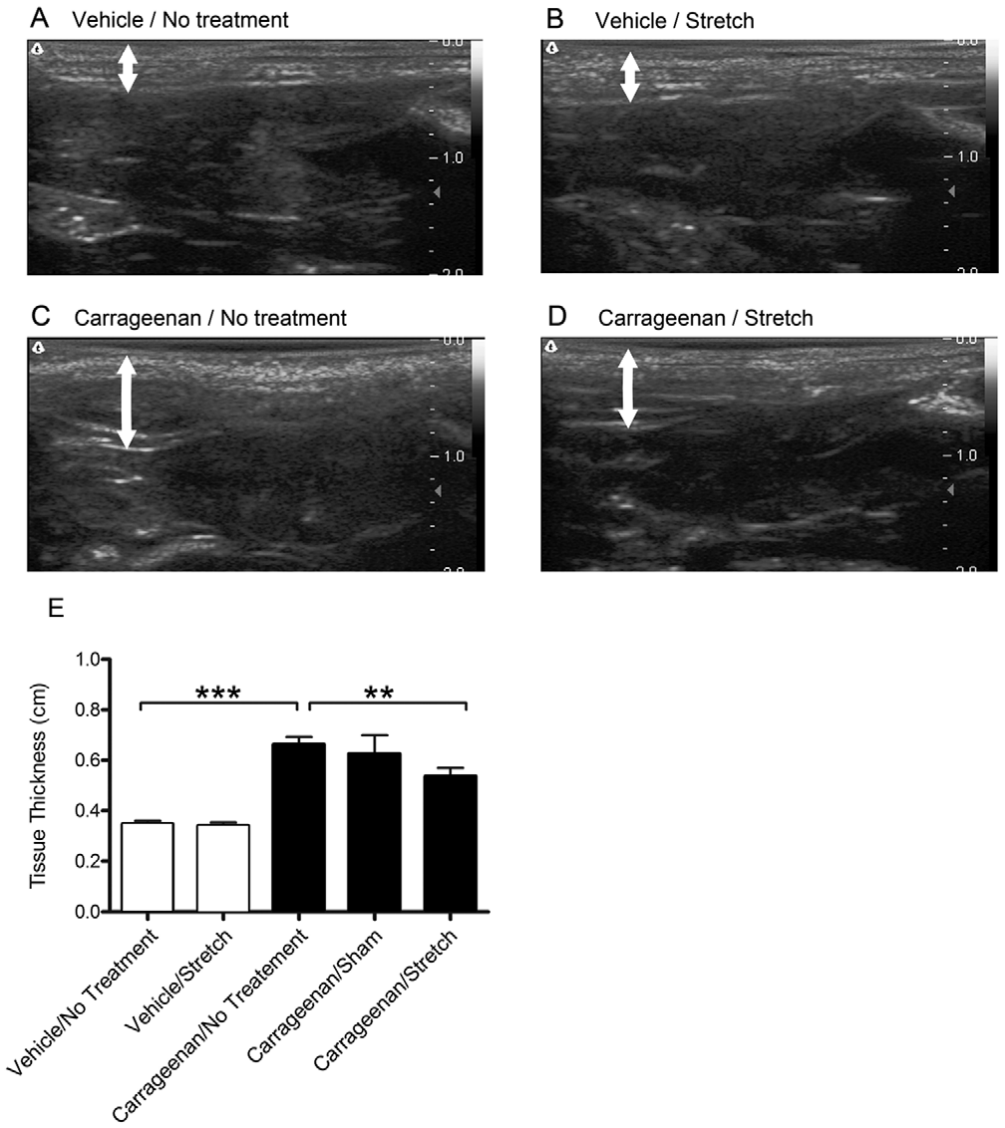
Ultrasound imaging and measurements. A–D: Examples of ultrasound images from vehicle/no treatment (A), vehicle/stretch (B), carrageenan/no treatment (C) and carrageenan/stretch (D) groups. White arrows indicate the location where ultrasound measurements of tissue thickness were obtained. E: Open and closed bars respectively represent the mean ± S.E tissue thickness (E) for vehicle and carrageenan groups. N = 4 Carrageenan/sham, N = 8 all other groups. (**p<.01, ***p<.001).

Ultrasound imaging allows for the visualization of changes in tissue structure *in vivo* (Figure 4 A–D). Inter-rater and intra-rater reliability coefficients (ICCs) for measurements of connective tissue thickness were r = .85 and r = .90 respectively, indicating that this technique was highly reliable for the rodent inflammation model. Average thickness of the tissues superficial to the deep back muscles was significantly different across experimental groups (p<.001). The carrageenan/no treatment group had increased thickness relative to the vehicle/no treatment group (p<.001; Figure 4E). Tissue stretch resulted in decreased tissue thickness measurements in the carrageenan/stretch group compared to the carrageenan/no treatment group (p<0.01) but not in comparison to the carrageenan/sham group (p = .12).

### Histology and Macrophage Expression

**Figure 5:**
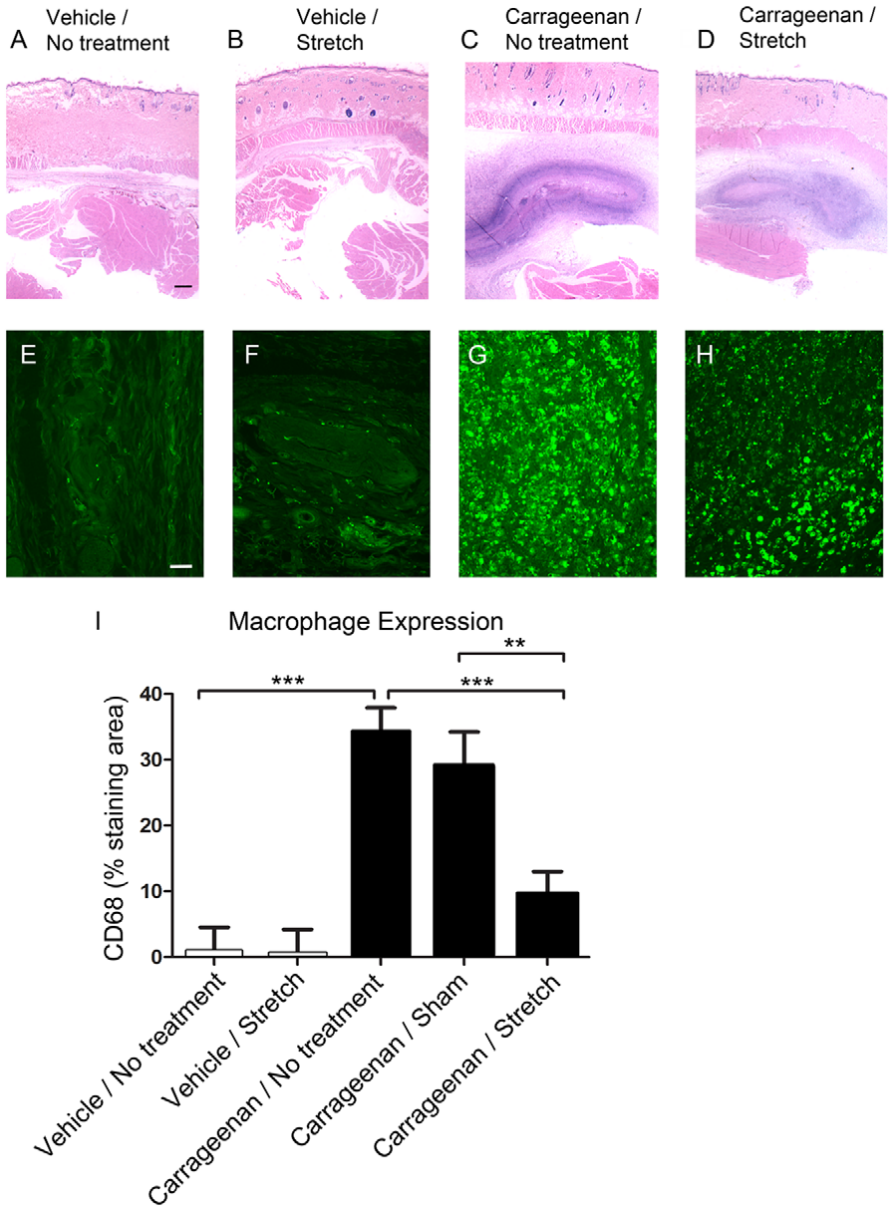
Histology and macrophage expression in the connective tissues of the low back. A–D: Representative images of Hematoxylin/Eosin staining of soft tissues from the lumbar region in a rodent in vehicle (A), vehicle/stretch (B), carrageenan (C) and carrageenan/stretch (D). Tissue structures from top to bottom (superficial to deep) include the dermis, pannicular muscle, nonspecialized connective tissues and the deep back muscles. E–H: Representative images of macrophage immunolabeling within the nonspecialized connective tissues from vehicle/no treatment (E), vehicle/stretch (F), carrageenan/no treatment (G), and carrageenan/stretch (H) groups. I: Macrophage expression quantified by calculating the percent CD 68 immunostaining area within each image. Bar graph represents means for each group ± S.E. Scalebars: black = 1 mm, white = 25 µm. Results are means = 4 carrageenan/sham, N = 8 all other groups. (**p<.01, ***p<.001).

H+E staining and CD68-immunoreactivity (IR) for macrophages were used to evaluate the peripheral inflammation associated with carrageenan injection. Figure 5A–D shows representative images of H+E staining from vehicle and carrageenan injected tissues. The carrageenan injection induced a predominantly monocytic infiltrate with abundant macrophages as well as lymphocytes and fibroblasts. In all cases, the inflammatory infiltrate was confined to the subcutaneous layer and did not penetrate the skin or underlying muscle. In a few cases, we observed mildly increased numbers of monocytes in the skin but not in the underlying muscle. In Figure 5E–H, CD68-immunoreactivity further demonstrates the presence of macrophages in response to inflammation in the nonspecialized connective tissues of the low back. Significant differences were detected across experimental conditions (p<.001; Figure 5I). A comparison of CD68 percent staining area showed an increase in macrophages in carrageenan compared to vehicle tissue. The carrageenan/stretch group had a substantially decreased macrophage expression in lumbar connective tissue compared to the carrageenan/no treatment group and carrageenan/sham groups (Figure 5I).

## Discussion

We found that carrageenan-induced inflammation of the nonspecialized connective tissues of the low back in the rat caused altered gait, increased local mechanical sensitivity and macrophage infiltration of connective tissues. All of these effects were ameliorated by tissue stretch. Our findings suggest that the nonspecialized connective tissues of the low back could be an important therapeutic target because: 1) inflammation of these tissues can cause pain and impair function and 2) the response of these tissues to a static stretch intervention could improve these outcomes.

The majority of animal models relevant to low back pain have focused on injury associated with the spine. Only a few musculoskeletal rodent models have considered the contribution of connective tissue structures to pain [13], [29], and no animal models so far have specifically examined inflammation of the nonspecialized connective tissues lateral of the spine. Our previous work identified and quantified calcitonin gene-related peptide (CGRP) expression in the nonspecialized connective tissues of the low back in control (no inflammation) animals, indicating a potential role for these tissues in the pathogenesis of pain. This finding was supported by the work of Tesarz et al., where CGRP and substance P innervation was quantified in the thoracolumbar fascia in the rodent [30]. In the present study we aimed to gain a greater understanding of the effects of inflammation within these nonspecialized connective tissues. A strength of the model is that it permitted us to evaluate two complementary behavioral measures: local measures of nociception (Von Frey testing) and global measures of movement impairment (gait). A limitation of mechanical sensitivity testing with Von Frey filaments is that they are applied to the surface of the skin and thus do not specifically target deeper tissues, while a limitation of using walking tracks to measure gait is that the velocity of each step cannot accurately be collected. To better understand whether altered gait was due to non-specific sickness behavior [31], [32] or anxiety [31], [33], either of which could affect gait velocity, we conducted an open field test and found no changes in overall activity, rearing, or time spent in the center of the field between experimental groups. The presence of gait abnormalities in our model suggests that the increased mechanical sensitivity detected with Von Frey testing was due to inflammation of deeper tissues (connective tissue and/or muscle) as opposed to simply the skin. Indeed, shortened stride length similar to that observed in our study was reported in a rodent model of “myofascial” inflammation produced by injection of paraformaldehyde into the multifidus muscles [29]. Although it is not possible to rule out sensitization of sensory afferents within muscle in the current study, we were able to document clear evidence of carrageenan-induced inflammation within paravertebral connective tissue that improved with stretch.

With the stretching technique used in this study, the animal was encouraged to hold a position of stretch that was slightly beyond its usual range of motion. This technique is relevant to active stretch therapeutic interventions (physical therapy, yoga) that involve slow and gentle, but non-habitual, body movements. Because our animal model involves a stretch of the whole, conscious animal, anti-inflammatory effects induced by stretching could involve central as well as local mechanisms. Centrally mediated effects of stretch could include stimulation of the hypothalamic-pituitary-adrenal axis and systemic cortisol secretion with direct anti-inflammatory effect on tissues. Another possibility is that stress during tissue stretch could have activated descending pain inhibitory pathways with inhibition of neurogenic inflammation via reduced secretion of neuropeptides (Substance P, CGRP) into the tissue. Future studies will attempt to differentiate between central and/or peripheral mechanisms that may underlie the improvements observed with stretch. A potentially important difference between our stretching method and stretching methods used in humans (e.g. yoga) is stress caused by the added restraint imposed by holding the animal by the tail. Further experiments comparing stretching in conscious vs. anesthetized rats will be necessary to further explore this issue.

Alternatively, or in addition, it is possible that stretching could have had a direct anti-inflammatory effect on the peripheral connective tissues of the low back. The direct response of cells and tissues to mechanical forces varies greatly depending on the manner in which the force is applied [34]. In cultured fibroblasts, repetitive or high amplitude cyclic stretch can lead to the production of pro-inflammatory cytokines [20] and apoptosis [35], whereas brief static stretch has been reported to decrease pro-inflammatory cytokines IL-3 and IL-6 [36]. Anti-inflammatory outcomes also were found in *in vitro* studies where low amplitude mechanical input was applied to chondrocytes [37], [38], [39], [40] and fibroblasts [41]. Other potentially relevant peripheral mechanisms involve the complex relationship between TGFβfibrosis and inflammation [42]. While repetitive or high amplitude mechanical input generally increases TGFβbrief static tissue stretch attenuated the increase in both soluble TGFβ-1 (*ex vivo*) and type-1 procollagen (*in vivo*) following tissue injury [19]. It is therefore plausible that a number of potentially interrelated local and systemic mechanisms may have contributed to the reduction in tissue inflammation observed in our *in vivo* animal model in response to stretch.

In conclusion, the role of the nonspecialized connective tissues in low back pain pathophysiology and treatment is not well understood but could involve localized inflammation. To address this question, we developed a rodent model of connective tissue inflammation and found that tissue stretch markedly improved both the local inflammation itself, as well as associated mechanical sensitivity and gait abnormalities. Further investigations using this model will be important to elucidate the mechanisms by which tissue stretch resulted in these therapeutic benefits.
